# Prognostic implications and diagnostic significance of TFE3 rearrangement in renal cell carcinoma

**DOI:** 10.1007/s00345-024-05290-w

**Published:** 2024-10-29

**Authors:** Carmina Muñoz Bastidas, Mario Tapia Tapia, Andrés Calva López, Vanessa Talavera Cobo, Juan Colombas Vives, Eduardo Miraval Wong, Cristina Gutiérrez Castané, Francisco Javier Ancizu Marckert, Marcos Torres Roca, Luis Labairu Huerta, Fernando Diez-Caballero Alonso, José Enrique Robles García, Felipe Villacampa Aubá, Daniel González Padilla, Bernardino Miñana López, Daniel Sánchez Zalabardo

**Affiliations:** 1 University of Navarra Clinic, Urology, Pamplona, Spain; 2 University of Navarra Clinic, Pathology, Pamplona, Spain; 3 University of Navarra Clinic, Urology, Madrid, Spain

**Keywords:** TFE3, TFE3-rearranged-RCC, Renal cell carcinoma, Prognosis, Fluorescence in situ hybridization

## Abstract

**Objectives:**

To investigate the impact of TFE3 rearrangement, analyzing clinicopathological features that influence renal cell carcinoma (RCC) recurrence, and clarify the role of immunohistochemistry (IHC) staining in diagnosis.

**Methods:**

We screened patients diagnosed of clear cell RCC (ccRCC), fluorescence in situ hybridization (FISH) was performed on all TFE3 positive IHC tumors. Clinicopathological and survival features were collected for analysis.

**Results:**

Out of 695 patients treated for renal tumors, 478 (68.7%) were ccRCC and 22 were suspected of TFE3 rearrangement based on IHC. Subsequent testing revealed 8 (1.15%) were positive in the FISH test (TFE3-rearranged-RCC) and 14 (2.01%) tested negative. No significant differences were noted in general characteristics among the three groups, except for age, TFE3-rearranged-RCC were younger than ccRCC (median age, 49 vs. 58 years, p = 0.02). TFE3-rearranged-RCC exhibited a significant higher recurrence rate compared to ccRCC (50% vs 18.8%) and multivariate analysis revealed that TFE3 rearrangement, along with tumor size and metastasis, was an independent prognostic factor for recurrence (HR = 4.6; 95% CI 1.1-21.2; p = 0.05). Survival analysis demonstrated a significant shorter PFS (progression-free survival) for TFE3-rearranged-RCC compared to ccRCC.

**Conclusions:**

TFE3 rearrangement is an independent prognostic factor for recurrence and contributes to a worse PFS, suggesting the necessity of careful follow-up. Diagnosis should be confirmed using FISH due to low specificity of IHC. Further studies are needed to confirm TFE3 IHC staining as a prognostic factor.

## Introduction

The understanding of the genetic mutations driving RCC carcinogenesis and its molecular classification has undergone significant evolution in recent decades. Initially designated as a distinct subtype in the 2004 WHO classification [1], Xp11.2 translocation/TFE3 gene fusion RCC was later reclassified in 2016 as part of the MiT (microphthalmia-associated transcription) family translocation RCC, alongside TFEB gene fusion RCC/t(6;11) RCC [2]. The MiT family comprises four genes—TFE3, TFEB, TFEC, and MiTF—each playing a unique role in regulating specific functions related to cell differentiation, autophagy, and lysosome generation [1, 3]. The 2022 WHO classification marks a significant advancement by introducing a molecular-based classification for renal tumors alongside the traditional morphology-based approach. This includes tumors such as TFE3-rearranged-RCC, TFEB-altered RCC, ALK-rearranged RCC, and ELOC mutated RCC [4].

Xp11.2 translocations result in the fusion of TFE3 with genes such as ASPL, PSF, and PRCC, leading to the overexpression of the TFE3 protein [3, 5]. Morphologically, TFE3-rearranged-RCC exhibits as a solid, brownish-yellow mass with a mixed papillary and nested pattern, with clear/eosinophilic cytoplasm [2, 6, 7], often accompanied by the presence of Psammoma bodies [6, 7]. TFE3-rearranged-RCC frequently exhibits heterogeneous morphological features, posing a challenge for pathologists in distinguishing them from other types [6]. While immunohistochemistry (IHC) remains a fundamental diagnostic tool, its accuracy may be compromised, given reported false positive rates and low predictive values [6, 8, 9]. FISH stands as the current gold standard for diagnosis despite its routine application is limited due to high costs [6, 10, 11]. Nonetheless, certain studies have concluded that while positive IHC TFE3 expression is not exclusive to the TFE3 rearrangement, it independently contributes to a more unfavorable prognosis, irrespective of the presence of the translocation [12, 13].

The incidence of TFE3-rearranged-RCC is low, accounting for 1–4% in adults and 20–75% in pediatric RCC [11, 14–16]. The prognosis remains controversial, ranging from similarity to ccRCC to the potential for rapid invasive disease [10, 12, 15–17].

The aim of this study is to assess the impact of TFE3 rearrangement by analyzing clinicopathological features that might affect RCC recurrence. Furthermore, we seek to explore how IHC staining can provide insights into this matter.

## Methods

### Patient selection and measures

We conducted a comprehensive review of 695 recorded adult patients with RCC who underwent either radical or partial nephrectomy at the University of Navarra Clinic from 2000 to 2023. Inclusion criteria comprised: (1) pathologically confirmed ccRCC; (2) RCC demonstrating positive TFE3 protein expression on IHC and (3) availability of complete clinicopathological information with a minimum follow-up of 6 months.

The primary study outcomes encompassed the presence of recurrence, progression-free survival (PFS), and overall survival (OS). Other variables collected included age, sex, tumor size, surgery type, surgical margins, TNM stage, positive TFE3 IHC, recurrence, and survival status. To confirm the diagnosis of TFE3-rearranged-RCC, FISH was performed on all cases with positive TFE3 IHC in the Department of Pathology.

### Immunohistochemical staining and evaluation

Tissue block sections embedded in paraffin and formalin-fixed of the renal cell carcinomas were immunohistochemically stained with the TFE3 (clone MRQ-37; Cell Marque) antibody. All samples were processed with BenchMark ULTRA IHC/ISH System (Ventana-Roche) automated staining platform and standard quality control procedures were carried out. The immunohistochemical staining of TFE3 was consider positive when there was moderate or intense nuclear staining.

### Fluorescence in situ hybridization assay

All immunohistochemically positive TFE3 renal cell carcinomas were analyzed with a FISH assay. We used a SPEC TFE3 dual color break apart probe (ZytoVision, Ref Z-2109) on 2 μm thick formalin-fixed and paraffin-embedded tissue sections. The signals of FISH were evaluated using a microscope Zeizz Axio Imager M2 by applying a triple-pass filter (DAPI/Green/Orange).

Signals were deemed split when the distance between orange and green signals was &gt;1 signal diameters. The TFE3 cases were FISH positive when the tumor samples contained more than 15% split signals.

### Statistical analysis

Statistical analysis of the data collected in the present study was conducted using SPSS version 20. Chi-square was employed for the statistical analysis of categorical variables, and the T Student test was utilized for quantitative variables. The Kaplan-Meier method and log-rank tests were applied to calculate OS and PFS, while Cox proportional hazard regression was employed for both univariate and multivariate analyses. Statistical significance was determined at a P-value less than 0.05.

## Results

Out of 695 patients treated for renal tumors, 478 (68.7%) were diagnosed with ccRCC, while 22 were initially diagnosed of TFE3 rearrangement based on IHC. Subsequent testing revealed that 8 (1.15%) were positive in the FISH test, confirming TFE3-rearranged-RCC, and 14 (2.01%) tested negative.

A comprehensive analysis of clinicopathological characteristics was conducted among the three groups (Table 1). Notably, patients with TFE3-rearranged-RCC tended to be younger, with a median age of 49 years, in comparison to ccRCC (*p* = 0.02).

**Table 1:** Patient and tumor characteristics of ccRCC and TFE3-rearranged and comparison between them

	ccRCC	TFE3-rearranged-RCC	RCC positive TFE3 HIC	*P*
	(*n* = 478)	(*n* = 8)	(*n* = 14)	
Age (years)*	58 (27–85)	49 (29–68)	53 (29–68)	**0.02**
**Sex**				**0.07**
Male	376 (79%)	4 (50%)	10 (71%)	
Female	102 (21%)	4 (50%)	4 (29%)	
Tumor size (cm)*	5.7 (1–21)	5.06 (1.7–10)	6.7 (2–13)	0.5
**Surgery type**				**0.01**
Partial nephrectomy	96 (3%)	5 (62.5%)	4 (29%)	
Radical nephrectomy	382 (80%)	3 (37.5%)	10 (71%)	
**Surgical margins**				0.2
Positive	15 (3%)	1 (12.5%)	1 (7%)	
Negative	463 (97%)	7 (87.5%)	13 (93%)	
**pTNM**				
pT1-T2	332 (69.5%)	4 (50%)	6 (42.9%)	0.2
pT3-T4	146 (30.5%)	4 (50%)	8 (57.1%)	
N0	437 (91.8%)	7 (87.5%)	12 (85.7%)	0.3
N+	29 (6.1%)	1 (12.5%)	2 (14.2%)	
M0	408 (85.4%)	6 (75%)	9 (64.3%)	0.3
M+	70 (14.65)	2 (25%)	5 (35.7%)	
**Recurrence**				**0.04**
Yes	90 (18.8%)	4 (50.0%)	4 (28.6%)	
No	388 (81.2%)	4 (50.0%)	10 (71.4%)	
**Survival status**				0.5
Cancer-related death	97 (20.3%)	1 (12.5%)	6 (42.9%)	
Alive or death from other causes	380 (79.7%)	7 (87.5%)	8 (57.1%)	
Follow-up (months) **	71.4 (1-275)	27.8 (9–78)	54.9 (7-238)	**0.001**

*Mean (range), ** median (range)

No significant differences were observed among the three groups in terms of sex, tumor size, surgery type, surgical margins and pTNM stage. However, TFE3-rearranged-RCC exhibited a higher recurrence rate than ccRCC (50% vs. 18%; *p* = 0.04), in spite of a significantly longer follow-up for ccRCC (71.4 months vs. 27.8 months; *p* = 0.001).

Univariate and multivariate Cox proportional hazard models were employed to elucidate the risk factors for recurrence (Table 2). The results of the multivariate analysis revealed that TFE3 rearrangement (HR = 4.6; 95% CI 1.1–21.2; *p* = 0.05) is as an independent prognostic factor associated with recurrence, in conjunction with tumor size (HR = 2.53; 95% IC 2.01–6.16 *p* = 0.006) and the presence of metastasis (HR = 4.36; 95% IC 2.86–7.39; *p* < 0.001).

**Table 2:** Univariate and multivariate analyses for variables for recurrence

	Univariate analysis			Multivariate analysis	
	HR (95% CI)	*P*		HR (95% CI)	*P*
Age	1.00 (0.98–1.02)	0.8		1.02 (0.99–1.03)	0.3
Sex (male)	0.83 (0.5–1.4)	0.5		0.91 (0.51–1.62)	0.7
Tumor size > 4 cm	3.52 (2.01–6.16)	**< 0.001**		2.53 (1.30–4.93)	**0.006**
Surgical margins	1.74 (0.60–5.08)	0.3		2.02 (0.51–8.03)	0.3
T-stage (T3-T4)	3.33 (2.11–5.25)	**< 0.001**		1.99 (1.14–3.47)	**0.01**
Metastasis	4.6 (2.86–7.39)	**< 0.001**		4.36 (2.44–7.79)	**< 0.001**
TFE3 IHC	1.72 (0.52–5.52)	0.3		0.58 (0.13–2.51)	0.5
TFE3 rearrangement	4.32 (1.05–17.56)	**0.04**		4.61 (1.10–21.20)	**0.05**

**Fig. 1:**
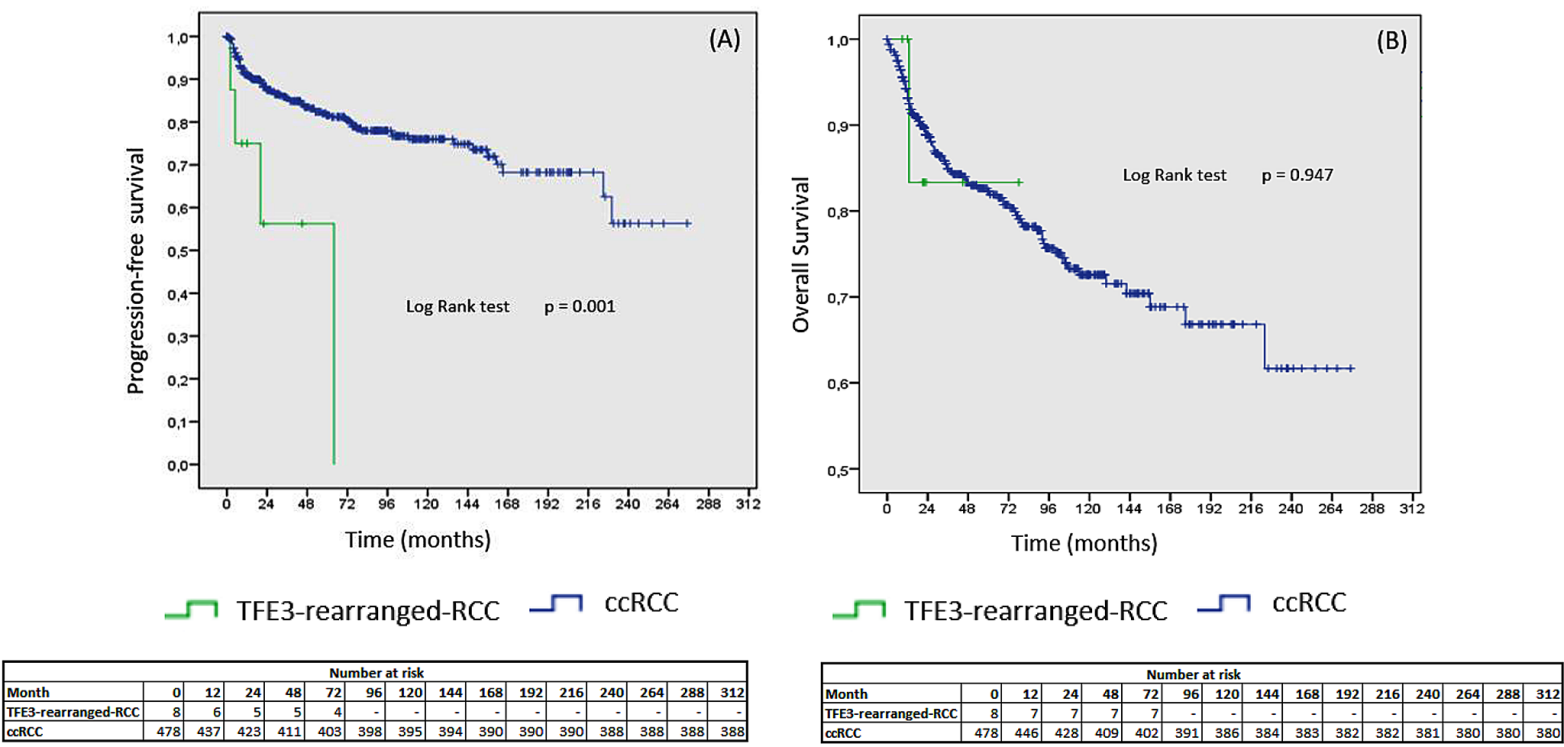
Kaplan-Meier curves. **(A)** Comparison of progression-free survival between TFE3-rearranged-RCC and ccRCC. **(B)** Comparison of overall survival between TFE3-rearranged-RCC and ccRCC

The results obtained from the survival analysis indicated that TFE3-rearranged-RCC was associated with a shorter progression-free survival compared to ccRCC (*p* = 0.001). As only one patient with TFE3-rearranged-RCC died from to cancer, there were no significant differences in the mortality rate and OS (Fig. 1. A and B).

## Discussion

TFE3-rearranged RCC is a distinct molecular subtype, as defined by the 2022 WHO classification [4], exhibiting a low incidence [14]. In our series, the incidence of TFE3-rearranged-RCC was 1.15% (8 out of 695 cases). A previous meta-analysis by Cheng et al. [14] highlighted a significantly higher incidence in females compared to males, with a pooled odds ratio (OR) of 5.13. Interestingly, survival features were found to be comparable between genders. TFE3-rearranged-RCC is characterized by the translocation of the Xp11.2 chromosome, which may explain the higher incidence in women. In contrast to some studies indicating gender-based differences [10, 11, 16–18], our results reveal an equal incidence in both men and women (50%/50%).

Since its recognition by WHO in 2004, the diagnosis of TFE3-rearranged renal cell carcinoma (RCC) relied on microscopic appearance and TFE3 IHC. However, several studies have demonstrated the limited accuracy of TFE3-IHC [6, 12, 13]. Klatte et al. [5] found that the positive predictive value of TFE3 immunostaining for TFE3-rearranged-RCC was only 12%, highlighting that IHC should not serve as a surrogate marker for diagnosis. Green et al. [9] conducted an immunohistochemical panel to identify consistently positive markers in these tumors, and some studies attempted combinations such as cathepsin K, HMB45, and others. However, the accuracy of these combinations did not reach a satisfactory level [6, 12, 13].

Lately, some studies have incorporated second-generation sequencing for the diagnosis of TFE3-rearranged RCC, as it can overcome the false negatives associated with FISH in specific fusion subtypes like NONO [10]. However, it’s important to note that these advanced sequencing techniques may not be universally available in all laboratories [6, 11, 12]. Currently, TFE3 break-apart FISH remains the gold standard for diagnosing TFE3-rearranged RCC [6, 11, 12]. In our current study, only 36% (8/22) of the TFE3 immunohistochemistry-positive samples were confirmed positive in the FISH assay.

According to previous studies, TFE3-rearranged RCC is more commonly diagnosed in young adults and has been suggested to exhibit aggressiveness comparable to ccRCC [10, 11, 16–18]. However, there is insufficient evidence to determine whether it independently contributes to lower PFS and OS. The current study revealed comparable tumor characteristics between ccRCC and TFE3-rearranged-RCC. Notably, patients with TFE3 rearrangement were significantly younger than those with ccRCC (median age, 49 vs. 58 years; *p* = 0.02) and demonstrated a markedly higher recurrence rate (50% vs. 18.8%; *p* = 0.04). This aligns with the findings of Lin et al. [12], supporting the notion that even with an early tumor stage at the initial diagnosis, recurrence and new metastasis are relatively common [12].

To our knowledge, this is the first study that compares TFE3-rearranged-RCC with ccRCC; survival analysis indicated that TFE3-rearranged RCC exhibited a significantly shorter progression-free survival, compared to ccRCC. However, given that only one patient with TFE3-rearranged-RCC died during the follow-up period, there were no significant differences observed in OS. The results of univariate and multivariate Cox proportional hazard models revealed that TFE3 rearrangement is an independent prognostic factor for recurrence, alongside tumor stage (HR = 4.6; 95% CI 1.1–21.2; *p* = 0.05).

Recent studies have highlighted that positive TFE3 IHC is linked to tumor progression and poor prognosis, regardless of the presence of TFE3 translocation [5, 10, 11]. Klatte et al. [5] conducted a reassessment of 75 RCC with morphological features suggestive of TFE3 translocation, revealing that 17 cases exhibited positive TFE3 IHC, while only 2 cases (2.6%) were genetically confirmed for the translocation through FISH or polymerase chain reaction (PCR). Their study demonstrated that positive TFE3 HIC had significantly worse disease-specific survival compared to RCC with negative HIC staining (HR: 3.3; CI 95% 1.03–11.1; *p* = 0.3). Notably, the presence of nuclear TFE3 immunostaining was associated with poor prognosis in the univariate analysis but not in the multivariate analysis [5].

We performed univariate and multivariate Cox proportional hazard models that suggest positive TFE3 IHC is not an independent prognostic factor for recurrence. On the other hand, Dong et al. [11], in both univariate and multivariate analyses, identified TFE3 positive IHC as an independent factor associated with poor progression-free survival (PFS). Lin et al. [12] found that both FISH-confirmed TFE3 rearrangement and positive IHC expression contribute to a poor prognosis in RCC. Our findings indicated that 42% of patients with RCC with positive TFE3 IHC died from cancer. Larger cohort studies are imperative to further delineate the implications of positive TFE3 immunostaining, not only as a screening marker for TFE3 rearrangement but also as a prognostic factor for recurrence and mortality.

However, it is crucial to acknowledge the limitations of our study. Firstly, its retrospective nature poses inherent constraints. Secondly, the relatively small population of TFE3-rearranged-RCC and the heterogeneity of the groups, given that ccRCC had a larger follow-up time, could introduce potential selection and information bias. Thirdly, RNA sequencing was not performed beyond the FISH assay, potentially impacting the accuracy of the diagnoses leading to classification bias.

In summary, TFE3-rearranged RCC represents a rare subtype, accounting for 1.15% of cases in the current study. The diagnosis of this subtype should be verified through FISH due to the IHC’s low specificity. Our study highlights TFE3 rearrangement as an independent prognostic factor linked to recurrence, leading to a compromised PFS. This underscores the importance of vigilant follow-up. Moreover, further investigations are warranted to validate TFE3 immunohistochemistry staining as a reliable prognostic indicator for recurrence and mortality.

## Data Availability

The data supporting our article’s findings are available upon reasonable request to the corresponding author.
